# REFINE special issue

**DOI:** 10.1007/s13346-022-01209-3

**Published:** 2022-07-26

**Authors:** Kathleen Spring, Klaus-M. Weltring, Adriele Prina-Mello, Ruth Schmid

**Affiliations:** 1 Gesellschaft fuer Bioanalytik Muenster e.V, Muenster, Germany; 2 Nanomedicine and Molecular Imaging Group, Clinical Medicine, Trinity Translational Medicine Institute (TTMI), Trinity College Dublin, St James’s Hospital, Dublin 8 Dublin, Ireland; 3 Laboratory for Biological Characterization of Advanced Materials (LBCAM), Trinity College Dublin, Dublin 8 Dublin, Ireland; 4 Trinity St James’s Cancer Institute, Trinity College Dublin, St James’s Hospital, Dublin 8 Dublin, Ireland; 5 Department of Biotechnology and Nanomedicine, SINTEF Industry, Trondheim, Norway

The number of new designs for medicinal products and medical devices that are based on nano(bio)materials (NBMs) is increasing rapidly and along such diverse lines that current regulatory testing will effectively become a bottleneck for innovation. The current regulation, initially designed for small chemical entities, is valid but its practical application to NBMs raises several analytical and experimental difficulties already in the preclinical assessment, which leads to a lack of confidence in the testing data. Regulators are aware of this danger and have started to get into a more intense dialogue with the scientific community and with developers of NBMs, to adapt the existing regulatory strategies. It is clear, that there will not be a disruptive replacement of the existing regulatory framework, but that there must be refinements to improve safety, cost/time efficiency, and sustainability. To support this refinement the partners of the REFINE consortium joint forces to pioneer a regulatory science framework (RSF) for the risk–benefit assessment of NBM-based medicinal products and medical devices.

This special issue presents approaches and work necessary to refine the RSF. The first basis for the refinement is the White Paper [1], where REFINE experts analysed and summarised the challenges associated with regulating nanotechnology-enabled health products published in available publications. The second basis was the analysis of the regulatory information needs for nanotechnology-based health products extracted from published regulatory documents [2]. These analyses revealed methods at early (pre-standardization) stages of development which have the potential to meet regulatory needs but also areas where currently no or unsuitable methods exist for regulatory assessment. Based on this analysis, it became clear that method development is necessary for two areas: (1) the interaction of NBM with cells, blood, and the immune system, and (2) the ADME and biodistribution of NBM.

Contributions to the first area are described in the articles Vandebriel et al. [3], Rijksinstitutt voor Volksgezondheid en Milieu, Tutty et al. [4] Trinity College Dublin, Perugini et al. [5] University of Brighton, Eder et al. [6] and Marzi et al. [7] Westfälische Wilhelms-Universität Münster. They analysed the possible interference of three model materials representing different classes in various in vitro assays. An important feature of the work described in these papers is the fact that the experimental protocols were adapted to NMBs and developed into standard operating procedures (SOP) by running them through interlaboratory round robins to bring the assays forward on their way to standardisation which is required to be accepted by regulators.

A big drawback in the regulatory assessment of NBM is the lack of information about the distribution of these materials in tissues and cells which would allow a better understanding of toxic effects and optimisation of physiologically-based pharmacokinetic modelling. Several publications in the special issue present complementary approaches to overcome this deficit. In vivo studies delivered data in Åslund et al. [8] SINTEF, were used to develop, improve, and verify a PBPK model of the biodistribution, which can be used to predict the absorption, distribution, metabolism, and excretion (ADME) of NBM in the future, described in Minnema et al. [9] Rijksinstitutt voor Volksgezondheid en Milieu and Montanha et al. [10] University of Liverpool. These approaches are complemented by the establishment of spheroid and organoid 3D cell culture systems to better study specific aspects of biodistribution such as penetration or accumulation of NBM in vitro down to visualisation at the subcellular level (Vennemann et al. [11] IBE R&D Institute for Lung Health gGmbH and 2 papers by Tutty et al. [12, 13]). The complementarity of these approaches enables researchers and NBM developers to better predict and thereby design NBM-based nanomedicines and medical devices due to a better understanding of the ADME of NBM.

A very practical tool to design NBM-enabled medical products and to estimate their possible toxic potential is the IT-based decision support system (DSS) developed under the responsibility of Green Decision and presented in Zabeo et al. [14]. The purpose of this system is to propose the most time- and cost-effective test strategy to cover the full preclinical characterisation of NMB-containing products. It consists of two modules focussing on the regulatory physicochemical characterisation assays for a given NBM and the testing of toxicological properties crucial for the regulatory examination of NBMs. The DSS will serve as a supporting tool for companies developing medicines and medical devices containing NMBs to meet regulatory demands.

Future regulation of NBM-enabled products will in the long-term be shaped by more harmonization across different application domains and between geographic regions to address harmonisation with other sectors. Similar challenges, knowledge gaps and regulatory questions across sectors as well as potential common initiatives need to be discussed with different communities as it is described in Halamoda-Kenzaoui et al. [15]. Joint Research Center European Commission. The need for such a cross-sectorial alignment of risk assessments of substances while taking into account the specificities of each sector (one substance-one assessment, *1S1A*) is highlighted by the new chemical strategy for sustainability. The approaches and results of the REFINE project described in this issue could serve as an example demonstrating how some objectives of the initiative 1S1A could be achieved and, at the same, time sector-specific differences addressed. Thereby, the REFINE project can serve as a blueprint for structural and straightforward development of regulatory science frameworks in different sectors as well as for successful interaction and exchange of information and experience within multi-sectorial communities.

Finally, an inspirational note by De Jong et al. Rijksinstitutt voor Volksgezondheid en Milieu [16], emphasizes the importance of the work compiled in this special issue towards regulatory-based, safe, and efficient NBMs in the market.

## References

[1] Halamoda-Kenzaoui B, et al. Perspective, opinion, or discussion. 2020;3(4). 10.33218/001c.13521.

[2] Halamoda-Kenzaoui B (2021). J Control Release.

[3] Vandebriel RJ, David CAW, Vermeulen JP, Liptrott NJ (2022). An inter-laboratory comparison of an NLRP3 inflammasome activation assay and dendritic cell maturation assay using a nanostructured lipid carrier and a polymeric nanomedicine, as exemplars. Drug Deliv and Transl Res..

[4] Tutty MA, Vella G, Vennemann A (2022). Evaluating nanobiomaterial-induced DNA strand breaks using the alkaline comet assay. Drug Deliv and Transl Res..

[5] Perugini V, Schmid R, Mørch Ý (2022). A multistep in vitro hemocompatibility testing protocol recapitulating the foreign body reaction to nanocarriers. Drug Deliv and Transl Res..

[6] Eder KM, Marzi A, Wågbø AM (2022). Standardization of an in vitro assay matrix to assess cytotoxicity of organic nanocarriers: a pilot interlaboratory comparison. Drug Deliv and Transl Res..

[7] Marzi A, Eder KM, Barroso Á (2022). Interlaboratory evaluation of a digital holographic microscopy–based assay for label-free in vitro cytotoxicity testing of polymeric nanocarriers. Drug Deliv and Transl Res..

[8] Åslund AKO, Vandebriel RJ, Caputo F (2022). A comparative biodistribution study of polymeric and lipid-based nanoparticles. Drug Deliv and Transl Res..

[9] Minnema J, Borgos SEF, Liptrott N (2022). Physiologically based pharmacokinetic modeling of intravenously administered nanoformulated substances. Drug Deliv and Transl Res..

[10] Montanha MC, Howarth A, Mohamed DA (2022). A physiologically based pharmacokinetic model to predict pegylated liposomal doxorubicin disposition in rats and human. Drug Deliv and Transl Res..

[11] Vennemann A, Breitenstein D, Tallarek E (2022). Subcellular detection of PEBCA particles in macrophages: combining darkfield microscopy, confocal Raman microscopy, and ToF–SIMS analysis. Drug Deliv and Transl Res..

[12] Tutty MA, Vella G, Prina-Mello A (2022). Pre-clinical 2D and 3D toxicity response to a panel of nanomaterials; comparative assessment of NBM-induced liver toxicity. Drug Deliv and Transl Res..

[13] Tutty MA, Movia D, Prina-Mello A (2022). Three-dimensional (3D) liver cell models - a tool for bridging the gap between animal studies and clinical trials when screening liver accumulation and toxicity of nanobiomaterials. Drug Deliv and Transl Res..

[14] Zabeo A, Rosada F, Pizzol L (2022). A Decision Support System for preclinical assessment of nanomaterials in medical products: the REFINE DSS. Drug Deliv and Transl Res.

[15] Halamoda-Kenzaoui B, Geertsma R, Pouw J (2022). Future perspectives for advancing regulatory science of nanotechnology-enabled health products. Drug Deliv and Transl Res..

[16] De Jong WH, Geertsma RE, Borchard G (2022). Regulatory safety evaluation of nanomedical products: key issues to refine. Drug Deliv and Transl Res..

